# An insight into the sialome, mialome and virome of the horn fly, *Haematobia irritans*

**DOI:** 10.1186/s12864-019-5984-7

**Published:** 2019-07-29

**Authors:** J. M. Ribeiro, Humberto Julio Debat, M. Boiani, X. Ures, S. Rocha, M. Breijo

**Affiliations:** 1 Section of Vector Biology, Laboratory of Malaria and Vector Research, National Institute of Allergy and Infectious Diseases, 12735 Twinbrook Parkway Room 3E28, Rockville, MD 20852 USA; 2 Instituto de Patología Vegetal, Centro de Investigaciones Agropecuarias, Instituto Nacional de Tecnología Agropecuaria (IPAVE-CIAP-INTA), Córdoba, Argentina; 3 Unidad de Reactivos y Biomodelos de Experimentación, Facultad de Medicina, Universidad de la República, Gral. Flores, 2125 Montevideo, Uruguay

**Keywords:** Vector biology, horn fly, malaria, virus, salivary glands, midgut, transcriptome

## Abstract

**Background:**

The horn fly (*Haematobia irritans*) is an obligate blood feeder that causes considerable economic losses in livestock industries worldwide. The control of this cattle pest is mainly based on insecticides; unfortunately, in many regions, horn flies have developed resistance. Vaccines or biological control have been proposed as alternative control methods, but the available information about the biology or physiology of this parasite is rather scarce.

**Results:**

We present a comprehensive description of the salivary and midgut transcriptomes of the horn fly (*Haematobia irritans*), using deep sequencing achieved by the Illumina protocol, as well as exploring the virome of this fly. Comparison of the two transcriptomes allow for identification of uniquely salivary or uniquely midgut transcripts, as identified by statistically differential transcript expression at a level of 16 x or more. In addition, we provide genomic highlights and phylogenetic insights of *Haematobia irritans* Nora virus and present evidence of a novel densovirus, both associated to midgut libraries of *H. irritans*.

**Conclusions:**

We provide a catalog of protein sequences associated with the salivary glands and midgut of the horn fly that will be useful for vaccine design. Additionally, we discover two midgut-associated viruses that infect these flies in nature. Future studies should address the prevalence, biological effects and life cycles of these viruses, which could eventually lead to translational work oriented to the control of this economically important cattle pest.

**Electronic supplementary material:**

The online version of this article (10.1186/s12864-019-5984-7) contains supplementary material, which is available to authorized users.

## Background

*Haematobia irritans* is a parasitic blood-feeding fly that spends most of its adult life in close contact with cattle, where they take small but frequent blood meals [1]. They stay mainly on the withers, back and side of the cattle, and at the belly during the hottest parts of the day. It belongs to the Muscidae family within the Brachycera sub-order, thus closely related to the non-blood feeding house fly *Musca domestica,* and being in the same tribe, Stomoxyini, of the blood feeding stable fly *Stomoxys calcitrans* [2]. Gravid adult females lay their eggs on cattle dung which serves as nutrition to their larvae [3]. It has a major economic impact on the cattle industry--estimated at several billion dollars per year [4–6].

Horn fly control is based on insecticides, which are applied when the infestation is massive [7]. Unfortunately, it has been reported resistance to many active ingredients such as pyrethroids, organophosphates or cyclodienes [8–10]. The emergence of resistance and the difficulty in developing new insecticides has triggered the search of innovative control tactics. Anti-vector vaccines have been proposed as alternate means of pest control, as exemplified by the anti-tick cattle vaccine based on a midgut antigen [11, 12]. Salivary vaccines targets have also been proposed both for vector control and parasite or virus transmission suppression [13–15]. In *H. irritans,* both approaches are being attempted for pest control: a transcriptome from adult flies aimed at identifying possible vaccine targets, including midgut targets [16], and recombinant salivary proteins are being tested as vaccine candidates [17–19]. Recently, a partial genome assembly of *H. irritans* became available and that might help further the discovery and rational selection of vaccine candidates [20].

Biological control is another alternative that is being investigated for control of livestock ectoparasites [21]. Adult and immature stages of horn fly have shown to be susceptible to entomopathogenic fungi [22, 23] while dung beetles reduced the survival of its larval stages [24, 25]. Pathogenic viruses were successfully used for controlling agricultural pests [26, 27]. However, literature associated with *H. irritans* viruses is negligible. There is only one study reporting the presence of a Nora-like virus, based on fragmented EST hits, on lab reared Mexican horn flies [28]. Recently, with the advent of inexpensive DNA sequence methods, the discovery of novel RNA viruses in vertebrate and invertebrate transcriptomes have led to an explosion in the discovery of new viruses [29].

In the present work we focused on a comprehensive description of the salivary and midgut transcriptomes of the horn fly, using deep sequencing achieved by the Illumina protocol, as well as exploring the virome of this fly. Comparison of the two transcriptomes allowed for identification of uniquely salivary or uniquely midgut transcripts, as identified by statistically differential transcript expression at a level of 16 x or more. A Densovirus and a Nora virus are described in detail.

## Results

### General information of the libraries and transcript assembly

After removing low quality bases as well as trimming remaining sequencing primers, two libraries made from the salivary glands yielded 190,725,362 and 221,725,784 reads, while two libraries from midguts yielded 104,485,166 and 122,836,125 reads of average length equal to 150 nt. Following assembly of these reads and extraction of 7,154 coding sequences, we selected 4,715 that were near full length and submitted their nucleotide mRNA and protein sequences to GenBank, which represents ~95% of the 4,977 protein sequences currently available for *H. irritans*.

We have mapped these 7,154 transcripts, summing 303,005 nucleotide bases (nt) to the recently published genome draft of *H. irritans,* which has a total of 4,521,647 nt [20]. We were able to successfully map all exons of 7.1 % of the sequences, amounting to 6.7% of the 7.154 transcript nucleotide bases. A total of 19.3 % of the transcripts accounting for 15.9 % of the transcript base count had at least one exon mapped but were incomplete.

Information regarding the 7,154 transcripts are available in Additional file 3. These are hyperlinked to several blast and rpsblast comparisons, which served to guide their functional annotation. The library reads were mapped to these transcripts and the number of reads accrued from each library, as well as the RPKM values for each transcript were calculated using the RSEM program. The average RPKM values according to the functional annotation of the transcripts from the SG and MG libraries are shown on Additional file 2: Table S1. Notice that the SG libraries indicate between 2-2.4-fold increased expression of transcripts associated with the transcription machinery, protein export machinery, and secreted classes, but the MG shows much larger expression (> 10-fold) of viral, immunity, and protein modification machinery (includes proteases), reflecting a higher diversity of the MG as compared to the SG tissues.

The program edgeR was used to identify the transcripts differentially expressed in the SG or MG, by selecting those that were significantly more expressed in either tissue. Additional file 1: Figure S1 shows the heat map of the transcripts that are differentially expressed, indicating the sharp delimitation between the two groups, and the larger complexity of the MG tissue.

### Transcripts overexpressed in the salivary glands

Additional file 2: Table S2 indicates the functional nature of transcripts that are overexpressed in the salivary glands of *H. irritans*. Their relative expression levels can be estimated by their E.I. values. The SG enriched transcripts were classified functionally as Secreted, Housekeeping, Transposable elements and Unknown. Among the secreted class are members of ubiquitously found protein families as well as transcripts coding for Stomoxyini specific families.

To help understanding of the classification of salivary protein families, follows a brief introduction to the role of saliva in blood feeding by insects, and *H. irritans* in particular: In order to feed on blood, hematophagous arthropods have to deal with the vertebrate hemostasis system, a redundant tripod of physiological responses consisting of platelet aggregation, blood coagulation and vasoconstriction [30, 31]. While most blood sucking insects contain one or more inhibitors for each of these three responses, salivary gland homogenates of *H. irritans* do not have anti-platelet of the apyrase class nor vasodilators, although anti-clotting activity has been found [32]. Salivary anti-inflammatory and immunomodulatory peptides have also been found in several blood feeding arthropods [33, 34]. Within *H. irritans*, the salivary peptide hematobin was found to inhibit macrophages [35].

### Ubiquitous protein families overexpressed in H. irritans salivary glands

#### Enzymes

Transcripts coding for endonucleases, serine proteases and lipases were found overexpressed in the salivary glands of *H. irritans*. Endonuclease expression in the salivary glands of sand flies and *Culex* mosquitoes have been acknowledge before, where they may decrease the inflammatory action of DNA released by damaged cells [36–38]. Endonucleases and serine proteases have been also described in the salivary transcriptome of *S. calcitrans* [39]. Serine proteases have also been found in the salivary glands of tabanids where they were implicated in thrombus degradation [40, 41]. Transcripts coding for proteins similar to lipases but also to yolk proteins [42] were found overexpressed in the salivary transcriptome of *H. irritans*. These contain a RGD domain that could potentially function as a platelet inhibitor [43], but could derive from fat body contamination of the salivary glands. While the expression index of the endonucleases is relatively high (5-10), those of the serine proteases (0.1-0.2) and lipases (0.6-0.7) are relatively low.

#### Immunity related transcripts

Transcripts coding for a cecropin and lysozyme were found overexpressed in the *H. irritans* sialotranscriptome. The *Haematobia* cecropin is distantly related (19.4% identity, 50% similarity) to the *S. calcitrans* protein named stomoxyn-2 [44, 45].

#### Small molecule binding domains

Lipocalins in triatomine bugs and ticks, and D7 proteins, related to the Odorant Binding Proteins (OBP), in mosquitoes and sand flies, help hematophagy by binding agonists of hemostasis and inflammation such as histamine, serotonin and leukotrienes [46]. The sialotranscriptome of *H. irritans* have contigs coding for two very similar lipocalin sequences similar to APO-D, having low expression indices of 0.022 and one contig coding for a member of the OBP family, that is moderately expressed with an E.I. equal 7.6. It remains to be determined whether this OBP member functions as a binder of agonists of hemostasis or is related to some chemical communication among flies via saliva.

#### Antigen 5

This is a ubiquitous protein family found in saliva of hematophagous arthropods and snake venoms. When known, their function is very disparate, as toxins in snakes [47, 48], as a superoxide dismutase in triatomine bugs [49], and as a IgG binder and possible complement activation inhibitor is *Stomoxys* [50]. In *Tabanus yao*, a member of this family acquired a disintegrins RGD motif that inhibits platelet aggregation by inhibiting the interaction of platelets with fibrinogen [51], and another incorporated a RTS domain and inhibits angiogenesis [52]. The contig named ab-48610_FR5_181-357 (Additional file 3) coded for a truncated member of the antigen 5 family, previously described as a strong *H. irritans* salivary antigen [53], and had collected the highest number of reads, thus with an E.F. value of 100. The full transcript was recovered when the abyss and the trinity assemblies were further assembled together (Additional file 1: Figure S1), indicating this strategy to be the best to recover full transcripts of highly expressed messages. The truncation appears to occur due to the inclusion of transcripts with non-removed introns that contain a stop codon. The phylogeny of the *H. irritans* deducted protein sequences of the antigen 5 family with their best matches from the Diptera database, plus the *Dipetalogaster maxima* protein known to have a superoxide dismutase activity is shown on Additional file 1: Figure S2. Three main clades are identified. The most abundantly expressed antigen 5 transcripts from *H. irritans* (ANO53937.1) is found in a subclade of clade I (named Ia in Additional file 1: Figure S2), closely related to *S. calcitrans* orthologs, while the more distantly related JAV16243.1, with a small E.I. value of 0.02, resides in clade III, together with a closely related *S. calcitrans* sequence. Interestingly, the highly salivary expressed proteins of this family have an alkaline pI above 8, while those of lesser expression have an acidic pI, similarly to mosquito salivary expressed antigen 5 proteins [54].

### Transcripts specific of the tribe Stomoxyini

#### Hematobin

A protein family named 15.6 kDa of unknown function was discovered following a transcriptome analysis of the salivary glands from *S. calcitrans* [39]. Several members of this family were discovered enriched in the salivary gland transcripts of *H. irritans*, one of which was characterized as a macrophage inhibitor [35] and is being evaluated as a vaccine target to control *H. irritans* load in cattle [19]. The contigs are well expressed, with E.I. values varying from 8 to 50. Phylogenetic analysis of the *H. irritans* members of this family, with their similar proteins found in GenBank indicates at least two major clades of this family occurring (Additional file 1: Figure S3), clade I having three subclades while clade II has two subclades. Hematobin (GenBank accession AJY26992.1) belongs to clade Ia, where the most distant member of the same subclade (ab-62535) has only 51% sequence identity and 68% similarity. Hematobin’ s identity to clade Ib members range from 40-46 % sequence identity, to clade Ic it ranges from 30-36% identity and to clade II members it is smaller than 30%. Psiblast of the Hematobin sequence against the NR protein database converges after nine iterations producing 122 matches, 106 of which are from insect species, including mosquitoes and fleas, with uncharacterized function. It appears that Hematobin belongs to a large family of insect-specific proteins.

#### Thrombostasin

The salivary anti-thrombin peptide from *H. irritans* has been previously characterized and names thrombostasin [55]. The assembled sialotranscriptome of *H. irritans* produced 55 contigs coding for proteins of this family, which contains also the orthologs of *S. calcitrans* [39]. Most of the *H, irritans* contig products of this family contain amino acid signatures indicative of furin cleavage sites [56, 57], suggesting the mRNA codes for a polyprotein that is further processed to produce the thrombin inhibitor. The *H. irritans* transcripts coding for thrombostasins are well expressed, reaching an E.I. value of 52.

#### Stomoxyini specific transcripts of unknown function

Additional file 2: Table S2 lists 11 transcript families that code for peptides of unknown function and are not found outside of Stomoxyini, or from outside of the genus *Haematobia*. Further information on these transcripts can be found in the Additional file 3. We highlight the family named “3.5 kDa alkaline salivary peptide” that has a relatively high E.I., averaging 45%, as well as the family “13.7 kDa alkaline salivary protein” with average E.I. of 13 %.

### Transcripts overexpressed in the midgut

To better understand the classification of midgut enriched transcripts, follows a brief introduction of the blood meal digestive process in *H. irritans* : The ingested blood meal by adult *Haematobia* is a very protein rich diet, requiring serine-type endoproteases and terminal carboxy and amino peptidases [58–60]. Glycosidases and lipases should exist as they are common in other insect digestive systems [61], but have not been characterized in *H. irritans*. A peritrophic matrix made of chitin and proteins envelops the blood meal [62, 63].

Additional file 2: Table S3 and Additional file 3 display the putative functional nature of 1,479 transcripts that are overexpressed in the midgut of *H. irritans*. Their relative expression levels can be estimated by their E.I. values. The MG enriched transcripts were classified functionally as “digestive enzymes”, “protease inhibitors”, “peritrophic matrix-associated”, “cytoskeletal”, “transporters and channels”, “immunity-related”, “putative secreted proteins with unknown function”, “detoxification”, “metabolism”, “transcription and translation”, “signal transduction”, “unknown”, and “viral products”. Some of these classes will be further analyzed below.

#### Digestive enzymes

Endopeptidases of the serine protease, metallopeptidase and threonine catalytic types were found overexpressed in the *H. irritans* midgut. The terminal peptidases aminopeptidases, carboxypeptidases and gamma-glutamyl hydrolase complete the peptidase suit of enzymes. Glycosidases, nucleotidases and lipases were also found enriched. Several of these enzymes contain a glycosylphosphatidylinositol (GPI) anchor close to their amino terminus, which attaches the secreted protein to the cell membrane, or microvilli.

Based on the best match to the Merops [64] database, 274 transcripts belong to the serine protease family of endopeptidases. These transcripts were clusterized based on their similarities at 75%, over 75% of their length, and then matched to the Merops database (Additional file 3). According to their best match to the Merops database, these proteases belong to the clans CG11864, CG14780, CG17571A, CG18493, CG3734, CG5233, CG5246, CG6041, CG6048, CG7142, CG7542, CG8299, CG9676, jonah, jonah 65Aiv, Try29F, Trypsin alpha, Trypsin lambda, Trypsin zeta, and Uncharacterized (Additional file 2: Table S4). Notice that within the same clan, there are cases of several groups of transcripts, indicative of genome duplication. Among members of the uncharacterized clan, there are sequences closely related to *Musca* and *Drosophila* annotated as lectizyme, and endopeptidases containing a leucine zipper indicating it may participate of signal transduction pathways or may target specific substrates. Most clans have more than one expressed transcript, indicating the multigenic status of these subfamilies. Many clans have expression indices higher than 50, namely Trypsin alpha a, Try29F c, and f and CG7542 a.

It is noteworthy that a transcript coding for apyrase was found enriched in the midgut. This enzyme cleaves phosphate from nucleotide di- and tri-phosphates, and has been previously described in hematophagous arthropods salivary glands where they destroy ADP and ATP released by platelets and neutrophils [65]. However, apyrases also were found expressed in the salivary glands of non-blood feeding *Anopheles gambiae* larvae [66], indicating this enzyme may serve as a terminal nucleotidase in Diptera and perhaps other organisms. This *Haematobia* enzyme does not have a predicted GPI anchor as it is common in terminal digestive enzymes, and it is possible that it prevents platelet or neutrophil aggregation/activation within the midgut contents.

#### Peritrophic matrix and mucins

*Haematobia irritans* adult flies have a thick peritrophic membrane enveloping the blood meal [63]. As indicated in Additional file 2: Table S3 and detailed in Additional file 1, there are 19 midgut enriched transcripts that have peritrophin (chitin binding) domains, some having up to 8 such domains, such as transcript tr-177214_FR6_2-427. Most of these transcripts also abound in serine or threonine residues in their carboxytermini that are identified by the program NetOglyc as putative mucin-type galactosylation sites. We also identified 37 transcripts that have 10 or more putative galactosylation sites, and we thus labelled them as putative mucins, which could have a role associated to the peritrophic matrix. Peritrophins have been proposed as vaccine targets [63], but their heavy glycosylation pattern may hinder vaccine effectiveness. Perhaps, targeting specifically the region of chitin-binding domains may be a best strategy.

#### Immunity-related products

Probably reflecting the potential bacterial growth in the midgut, 57 transcripts associated with pathogen pattern recognition function and antimicrobial activity were found over expressed in the midgut when compared to the salivary glands. Transcripts coding for tyrosinase inhibitors were additionally included in the class. These transcripts code for a peptide that is 75 % identical to the phenol oxidase inhibitor found in the hemolymph of *Musca domestica* [67]. They may modulate immune-mediated phenol-oxidase activity, or if secreted to the hemolymph, they may regulate cuticle melanization as proposed before [67].

Transcripts coding for antimicrobial polypeptides of four different families (attacin, cecropin, defensin and lysozyme families) were found enriched in the midgut transcriptome. The defensin-coding transcripts were relatively well expressed, with E.I. values ranging from 10 to 35.

#### Putative secreted products of unknown function

Additional file 2: Table S5 lists over 300 transcripts that code for putative secreted polypeptides that are overexpressed in the midgut. They include transcripts coding for proteins having similarities to known products that have unknown function and those that appear to be unique to Stomoxini or to *Haematobia* and have no known function. Many of these transcripts have high expression levels, as indicated in Additional file 2: Table S5. It is possible that many of these may have antimicrobial activity. These transcripts can be found further classified in Additional file 1.

#### Transporters and ion regulation

Transcripts coding for several transporters are found enriched in the midgut of *H. irritans* (Additional file 2: Table S6). These not only include those associated with amino acid and glucose transport, but also those associated with the gut alkalization, such as V-ATPase subunits and associated carbonic anhydrase [68–71].

#### Lipid binding proteins

Possibly associated with the transport of lipids intracellularly and their export to the hemolymph, various transcripts coding for proteins with lipid binding domains are found enriched in the midgut transcriptome. These transcripts are characterized by coding for two different members of the JHBP (juvenile hormone binding proteins) family and lipocalins of the Apo-D and cytosolic fatty-acid binding protein families (Additional file 3).

#### Lipid metabolism

Transcripts coding for Acyl-CoA synthetase, acyl-CoA-binding protein, very long-chain fatty acid CoA synthetase, ecdysteroid kinase, lipases, lipid exporter ABCA1, peroxisomal acyl-CoA oxidase, phosphatidylinositol transfer protein SEC14, serine palmitoyltransferase, fatty acid hydroxylase, and lipid phosphate phosphatase were found enriched in the midgut and are probably associated with lipid digestion and transport.

#### Cytoskeletal proteins

The mosquito midgut presents dramatic changes in ultrastructure following a blood meal, which is accompanied by expression of specific cytoskeletal proteins [72]. The midgut of blood feeding *Haematobia irritans* similarly expresses significant large amounts of transcripts coding for members of the innexin, actin, drebrin, dynein, myosin, and troponin families, reflecting the contribution of smooth muscles associated with this organ and not with the salivary glands.

#### Other midgut overexpressed transcripts

Additional file 1 displays other midgut enriched transcripts, including those associated with detoxification, amino acid metabolism, carbohydrate metabolism, energy metabolism, intermediary metabolism, nucleotide metabolism, protein modification, proteasome machinery, protein synthesis machinery, signal transduction, transcription machinery, unknown conserved, and unknown. We highlight the presence of two transcripts coding for the neuropeptides CCHamide-2 and Neuropeptide-F, both of which have been implicated in the feeding physiology of *Drosophila* [73–78]. Several of the transcripts without a known function have relatively high expression indices.

### Viral discovery

The transcriptome assembly of *H. irritans* was subjected to Blastx searches (E-value <1e-5) against a reference virus proteins database. Eleven transcripts showing similarity to Nora viruses (E-value = 1e-31 to 0) and eight similar to densoviruses (E-value = 1e-09 to 0) were found.

After curating of the Nora-like transcripts by cycles of read mapping and *de novo* assembly, a highly supported virus sequence of 12,002 nt was re-assembled (mean coverage 5,684X, total virus reads 454,873). Sequence annotation indicated the presence of four ORFs flanked by a 281 nt 5’UTR and a large 465 nt 3’UTR followed by a Poly(A) tail (Additional file 1: Figure S4.A). Sequence alignments of the obtained sequence and its predicted gene products indicated similarity with Nora viruses, and highest identity (61.7% at the nt level and between 34.1 to 73.2% of the predicted proteins) with *Drosophila subobscura* Nora virus (GenBank KF242510) [79] and to a similar extent to other *Drosophila-*isolated Nora viruses [80–82]. Further, comparison of the detected sequence with the reference sequence of Nora virus (NV, GenBank NC_007919) at both the nt and aa levels resulted in equivalent identity levels and a common genomic architecture (Additional file 1: Figure S4.C). In this scenario, we tentatively proposed that the obtained sequence corresponded to a novel virus which could be member of a new species which we dubbed *Haematobia irritans* Nora virus (HiNV, strain URU). To entertain this hypothesis, we moved forward to thoroughly annotate and generate evolutionary insights of HiNV. ORF1 of HiNV-URU (coordinates 282-1,805) encodes a 507 aa 59.3kDa protein, sharing 35.9% aa identity (AI) with VP1 protein of NV, which is a viral silencing suppressor (VSR) [79]. Overlapped with ORF1, ORF2 extends between coordinates 1,768-7,929 nt encoding a large VP2 protein (2,053 aa – 233.9kDa), presenting typical domains of Nora virus replicases. VP2 has three trans-membrane sites at its N-terminal region, followed by a viral helicase domain (HEL, pfam00910, E-value = 5.4e-11), a serine protease (PRO, HHPred id: 2HAL_A, E-value = 8.3e-10, probability 98.64%), and at the C-region an RNA dependent RNA polymerase domain (RdRP, pfam00680, E-value = 1.25e-38). The VP2 of HiNV-URU shares an overall 52.2% AI with NV, but AI extends as high at 72.8 and 74.6% at the HEL and RdRP domains, suggesting a selective pressure acting asymmetrically along the protein to conserve its functional domains and thus its putative activity. Overlapped with ORF2, ORF3 (7,913-8,743 nt coordinates) encodes a 276 aa 31.5kDa protein, with 30.2% AI with NV VP3, the most divergent protein of the virus. HiNV-URU VP3 is structurally similar to the outer capsid protein sigma-1of orthoreoviruses (OCP, HHPred id: 6GAP_A, E-value = 8.9e-10, probability 99.07%). Finally, ORF4 (8,859-11,537 nt coordinates), encodes a coat protein of 892 aa and 98kDa, sharing a 71.1% AI with VP4 of NV and presenting the typical subunit structural domains VP4C-VP4B-VP4A observed in the cryo-em structure determined for NV (RCSB PDB: 5MM2, probability 100%, E-values 1.5e-93 (VP4B), 6.6e-107 (VP4C) and 9.2e-143 (VP4A). All in all, HiNV-URU appears to have the genomic architecture of a Nora virus (Additional file 1: Figure S4.C). The *Drosophila* Nora virus has been shown as an enteric virus [83], mostly found in the intestine of infected flies, which show increased vacuolization upon infection. NV is then excreted in the feces and is horizontally transmitted. Interestingly, when exploring our datasets, we observed very high relative RNA levels of HiNV in our midgut libraries, reaching 6,825 reads per million of total library reads (RPM), and negligible levels of virus RNA in the salivary glands: 2-21 RPM (Additional file 1: Figure S4.B, Additional file 2: Table S9). This indirect evidence supports the likelihood that HiNV might share the biology and mode of transmission of NV in flies. Nevertheless, additional experiments should asses this possibility. In addition, Torres et al [28] reported the presence of a Nora virus, based on fragmented EST hits of lab reared Mexican horn flies. We retrieved those ESTs (GenBank HO004689, HO000459, and HO000794) and reconstructed a partial region of a VP4 CDS which shared between 82.5 to 85.2% nt identity with HiNV-URU. Thus, we believe the flies described by Torres et al [28] presented a strain of HiNV which we dubbed here as HiNV-MEX. We then assessed whether HiNV might be present in additional *H irritans* high-throughput datasets. We found 26 additional public libraries, and interestingly in two of them, corresponding to lab reared horn flies from Saint Gabriel, LA, USA, we found evidence of HiNV RNA (Additional file 2: Table S9). Given the high number of virus reads we were able to reconstruct, with robust support, the complete genome of a virus sequence which we dubbed HiNV-USA, which is 11,985 nt long (mean coverage 2,389X, total virus reads 530,284). HiNV-USA shares an 82.7% nt identity and their predicted gene products have a 29.6% (VP3) to 93.8% (VP4) AI with HiNV-URU. To assess the evolutionary landscape of HiNV we generated multiple capsid protein alignments of the three putative strains of HiNV and that of diverse Nora like viruses. We observed that the VP4 of HiNV lacks a short C-region of the protein, which is highly conserved in Drosophila Nora viruses, but missing in other insect Nora viruses (Additional file 1: Figure S4.E). We interrogated our dataset and confirmed that the premature end of translation of HiNV VP4 CDS was significantly supported by virus reads, and thus appears not to be artifactual or a result of poor assembly (Additional file 1: Figure S4.F). We used these multiple alignments as input to generate maximum likelihood phylogenetic trees. Our results unequivocally cluster the three HiNV putative strains in a separate sister clade to *Drosophila* Nora viruses and a Nora like virus associated to bees (Additional file 1: Figure S4.D). HiNV was well within the Nora clade, which shows moth and parasitoid wasps associated Nora like viruses as more divergent, and perhaps could be placed in separate genera. The discovery of additional Nora viruses and hosts could be useful to elucidate the evolutionary history of this highly diverse clade of viruses, which has not been formally classified by the International Committee on Taxonomy of Viruses (ICTV) yet. Moreover, it remains obscure whether any pathogenic effect could derive of HiNV infection in horn flies, which could eventually lead to the development of control strategies based on viruses (virocontrol) of this important cattle plague.

We then returned to our transcriptome hits of densovirus like transcripts. After curating by iterative read mapping and *de novo* assembly, a highly supported virus sequence of 4,283 nt was re assembled (mean coverage 43,751X, total virus reads 1,249,238). Sequence annotation indicated the presence of four ORFs flanked by an 81 nt 5’UTR and a 181 nt 3’UTR (Additional file 1: Figure S5.A). The predicted products of the largest ORFs (dubbed NSP1 and VP1) shared 32.7 and 38.5% highest AI with the non-structural protein 1 and the VP1 of Linvill Road Virus (LRDV, GenBank AQN78650.1) which was recently isolated from *Drosophila*. These proteins shared comparable values of similarity to the non-structural protein 1 of the moth-isolated *Dendrolimus punctatus* densovirus (GenBank YP_164339.1) [84] and to the structural protein VP1 of *Culex pipiens* densovirus (GenBank YP_002887627.1) [85]. Both viruses are proposed to belong to families known to infect invertebrates [86, 87]. Densoviruses are ssDNA genome viruses from family *Parvoviridae,* which have been proposed as insect genome transformation tools [88]. In this context and given that we reconstructed our sequence based on RNA data, we tentatively suggested that this complete coding (CC) sequence corresponds to a new virus, which could be a member of a novel species, which we named *Haematobia irritans* densovirus (HiDV). We then proceeded to further annotate and explore this virus sequence. ORF1 (82-1,983 nt coordinates) encodes a 633 aa 74.3 kDa protein which contains a parvovirus non-structural protein NS1 domain at its C-terminal region (Parvo_NS1, cl24009, E-value = 3.33e-09) which is essential for DNA replication (Cotmore et al., 2019). Within the NS1 CDS there is an additional overlapped ORF which encodes a 274 aa 31.3 kDa protein of unknown function (HP1). Interestingly, while this ORF has not been annotated in the similar LRDV, Tblastn searches using as query the HiDV HP1 showed that this protein appears to be conserved and equilocal in both viruses (E-value = 2e-16, AI 42%). We presume then that HP1 (255 aa 28.5 kDa in LRDV) might be relevant for the virus. After a short AT rich 55 nt long spacer region a second large ORF is present in HiDV (VP CDS, 2,039-4,102 nt coordinates), encoding a 687 aa 76.8 kDa structural protein. VP1 presents in the N-terminal region a Parvovirus coat protein N domain (Parvo_CP, pfam08398, E-value = 4.90e-15), followed by a Capsid protein VP4 domain (Denso_VP4, pfam02336, E-value = 1.58e-03). Within this structural encoding CDS, we found an additional overlapped short ORF predicted to encode a 149 aa 17.1 kDa protein (HP2). We failed to retrieve any similar protein in other viruses, but again, HP2 is similar to an unannotated ORF at equilocal position in LRDV, which generates a 111 aa protein which shares a 53% AI with HiDV HP2 at the C-terminal region. To investigate a tentative tropism of HiDV based on RNA data, which could suggest viral mRNA expression derived from infection, we explored our datasets and found out that virus RNA was highly enriched in the midgut libraries, reaching more than 2% of total RNA in one of the samples and almost negligible levels in salivary glands (Additional file 1: Figure S5.C). We then assessed whether HiDV was present in additional public *H. irritans* high-throughput public datasets. Interestingly, we found evidence of HiDV in five other samples from two studies from horn flies from USA (Additional file 2: Table S9), (BioProject PRJNA30967 and PRJNA429442). In the latter study, with 29,484 HiDV derived reads, we were able to explore the genetic diversity of these viruses’ sequences. Unlike HiNV, where strains detected in horn flies from Uruguay differed as high as 18% at the nt level with HiNV-USA, HiDV from horn flies isolated in USA differed by only 65 variable sites (P-value <1e-12, less than 1% overall nt divergence), mostly single nucleotides polymorphism when comparing with HiDV from Uruguay horn flies. These polymorphisms were detected with significant support ubiquitously along the genome (Additional file 2: Table S8). An important share of the observed variants is silent, but some generate aa substitutions on the respective gene products (Additional file 1: Figure S5.D). We then generated phylogenetic insights based on multiple sequence alignments of HiDV and densoviruses refseq VP proteins. The obtained trees showed that HiDV clusters within the *Densovirinae* subfamily of parvoviruses (Additional file 1: Figure S5.B) [86]. Local topology within *Densovirinae* shows that HiDV branches with LRDV, basal to a clade of unassigned densoviruses linked to other invertebrates, ambidensoviruses and iteraviruses (Additional file 1: Figure S5.E). Additional related viruses are needed to comprehend the evolutionary trajectory of these viruses. It is worth noting how little we still know about the viruses of horn flies and related insects. The viruses presented here are only a first glance of the *H. irritans* virome.

## Discussion

While most transcriptomic studies focus primarily in a single organ or tissue, in this work we analyzed simultaneously two transcriptomes from the cattle ectoparasite, *Haematobia irritans*. Illumina reads from the salivary glands and midgut were “de novo” assembled, the coding sequences extracted, and the reads from each library mapped to these CDS. Statistical tests indicated the transcripts that were significantly overexpressed in each tissue. Further selection of these transcripts that were at least 16-fold overexpressed in either organ led to a salivary-enriched and a midgut-enriched set of transcripts. These transcripts are a mining field for anti-*Haematobia* vaccine development. One of the salivary transcripts have already been used for this purpose [19, 53].

A tick midgut antigen named BM86, containing a GPI anchor, has been successfully used as a vaccine to control the cattle tick, *Rhipicephalus microplus* [12]. However, similar approaches to control insect pests have been unsuccessful. Two major differences exist between ticks and blood feeding flies regarding their digestion mechanism: Tick midgut cells ingest blood by pinocytosis and an intracellular digestion, mainly done by lysosomal cathepsins, proceeds; flies secrete serine proteases into the midgut that cleaves blood proteins in smaller oligopeptides, which are further digested by microvilli-associated amino and carboxy-peptidases. This indicates that the blood bolus in tick midguts are relatively undisturbed, while in blood feeding flies the blood meal, including antibodies, suffers attack by the digestive enzymes [60]. Hematophagous flies also have a much thicker peritrophic matrix that functions as a dialysis membrane preventing larger molecules, such as hemoglobin and immunoglobulins, to diffuse out of the enveloped meal [62]. Accordingly, compared to ticks, *Haematobia* anti-midgut vaccines should be more difficult to develop. Notwithstanding these difficulties, midgut peritrophins, which are components of the peritrophic matrix, have been proposed as vaccine targets [63], but results were inconclusive. Perhaps a two-antigen approach could be tried: Component (1) would be a peritrophin vaccine that disrupts or delays the peritrophic matrix formation, while component (2) would target a membrane bound antigen. The set of midgut enriched protein sequences described in this paper contains various peritrophins and GPI-anchored proteins that could serve as candidate antigens for trying this approach. This strategy should be more effective in the first blood meal of flies, when the peritrophic matrix is not formed yet.

The use of viruses for pest control is exemplified by the baculovirus products aiming at lepidoptera larval control [89], and by natural epizootics of viruses affecting insect populations (reviewed in [26]). Recently, with the advent of inexpensive DNA sequencing methods, the discovery of novel viruses have exploded [29]. Very frequently RNAseq experiments from vertebrates, invertebrates and plants uncover novel RNA viruses, within the context of meta-transcriptomics [90]. Here we report two novel viruses infecting *Haematobia irritans*. While these viruses may not be pathogenic to the fly, they may contribute to the molecular tool box that one day may lead to the design of pathogenic viruses (for example, the described viruses have the proper cell invasion and replication machineries to survive within *Haematobia*). As the virome of insects increases, it may be possible for a fly virus of another Muscidae or Brachycera to be infective and pathogenic to *Haematobia*.

## Conclusion

We provided in this work a comprehensive catalog of 7,154 transcripts and their protein sequences associated with the salivary glands and midgut of the horn fly. The majority (92%) of these proteins have no matches to the publicly available partial genome of *H. irritans* [20], thus being a valuable resource in identifying proteins by mass spectrometry and for screening for vaccine candidates. Additionally, we discover two midgut-associated viruses that infect these flies in nature. Future studies should address the prevalence, biological effects and life cycles of these viruses, which could eventually lead to translational work oriented to the control of this economically important cattle pest.

## Methods

### Insects

Horn flies were captured from naturally infected cattle of Campo Experimental, Instituto de Higiene, Facultad de Medicina, Canelones, Uruguay (34 38’ S, 55 55’ W), following license number 071140-000611-10 from the Institutional Animal Care and Use Committee (IACUC) of the Universidad de la República, Facultad de Medicina. The flies were anesthetized by placing them at 4 °C for 5 minutes and fixed with an insect pin to a silicone matrix (Sylgard™). Under a binocular stereomicroscope horn flies were dissected, and the salivary glands and the midguts extracted. A total of 100 glands and 50 midguts per sample were directly placed in cool TRizol™ (Invitrogen). Samples were obtained between December 2015 and February 2016.

### RNA preparation

Total RNA from salivary glands and midguts were extracted using a RNeasy mini total RNA isolation kit (Qiagen, USA), according to the manufacturer’s protocol. The samples of purified RNA were placed in GenTegra® tubes following the manufacturer protocol and shipped at room temperature for further processing.

### DNA library construction and sequencing

This was done as reported before [91]. Briefly, RNA quality was assessed by Agilent 2100 Bioanalyzer with an RNA 6000 Nano Chip (Agilent Technologies, USA). The NEBNExt Poly(A) mRNA Magnetic Isolation Module (New England Biolabs, USA) was used to purify the mRNA using oligo-dT beads. The NEBNext Ultra Directional RNA Library Prep Kit (NEB) and NEBNext Mulitplex Oligos for Illumina (NEB) were used to construct complementary DNA (cDNA) libraries for Illumina sequencing. The libraries were sequenced in an Illumina HiSeq 2500 DNA sequencer, utilizing 125 bp single end sequencing flow cell with a HiSeq Reagent Kit v4 (Illumina, USA).

### Bioinformatic analysis

Bioinformatic analyses were conducted following the methods described previously [92, 93], with some modifications. Briefly, the fastq files were trimmed of low quality reads (<20), contaminating primer sequences were removed. The clean reads were concatenated for single-ended assembly using the Abyss [94] and Trinity [95] assemblers. The resulting assemblies were further assembled using a iterative blast and CAP3 pipeline [96]. Coding sequences (CDS) were extracted based on the existence of a signal peptide in the longer open reading frame (ORF) and by similarities to other proteins found in the Refseq invertebrate database from the National Center for Biotechnology Information (NCBI), proteins from Diptera deposited at NCBI’s Genbank and from SwissProt. Reads for each library were mapped on the deducted CDS using blastn with a word size of 25, 1 gap allowed and 95 % identity or better required. We use the “expression index” (EI) to compare transcript relative expression among contigs, defined as the number of reads mapped to a particular CDS multiplied by 100 and divided by the largest found number of reads mapped to a single CDS. Functional classification of the transcripts was achieved by scanning the different blast and rpsblast results. Classification of the proteases and protease inhibitors were based on the transcript blast matches to the Merops database [64].

Protein alignments were done using ClustalX [97]. Phylogenies were inferred using the Mega6 package [98], using the Neighbor-Joining method [99]. The evolutionary distances were computed using the Poisson correction method [100] and are in the units of the number of amino acid substitutions per site. The rate variation among sites was modeled with a gamma distribution (shape parameter = 1). Transcript translations were clustered according to their similarities over at least 75% of the larger sequence; the clusters being mapped to Additional file 3, including links to the sequences of the cluster in fasta format, as well as their clustalX alignments. Heat plots were made with the package gplots [101] from the R package [102]. Statistical analysis was done with the package edgeR [103].

Virus discovery, genome assembly and annotation, and evolutionary insights were conducted as described in [54, 104]. In brief *de novo* transcriptome assemblies were explored by BLASTX searches (E-value = 1e-5) against a refseq of viral proteins database available at ftp://ftp.ncbi.nlm.nih.gov/refseq/release/viral/viral.2.protein.faa.gz. The resulting matches were annotated by iterative mapping as described elsewhere [105]. The resulting sequences were used as input for ORFs prediction by ORFinder available at https://www.ncbi.nlm.nih.gov/orffinder/. Functional and structural domains of the predicted gene products were annotated using standard tools (NCBI CDD https://www.ncbi.nlm.nih.gov/Structure/cdd/wrpsb.cgi and HHPred https://toolkit.tuebingen.mpg.de/#/tools/hhpred0. TMHMM 2.0 was employed for transmembrane predictions (http://www.cbs.dtu.dk/services/TMHMM-2.0/). Abundance of virus RNA was calculated as reads per million (RPM) by mapping with standard parameters using Bowtie2 http://bowtie-bio.sourceforge.net/bowtie2/index.shtml. Phylogenies were generated based on multiple alignments of capsid (CP) proteins using MAFFT v7 https://mafft.cbrc.jp/alignment/software/ with the BLOSUM64 scoring matrix for amino acids and G-INS-i iterative refinement method. Uninformative sites were trimmed using GBlocks tool v.0.91b (http://molevol.cmima.csic.es/castresana/Gblocks_server.html). Maximum likelihood phylogenetic trees were generated with FastTree v2.1http://www.microbesonline.org/fasttree with JTT-CAT models of amino acid evolution, 1000 tree re-samples and local support values estimated with the Shimodaira-Hasegawa test. Polymorphisms were detected using the Freebayes tool with standard parameters (https://github.com/ekg/freebayes/blob/master/README.md) and visualized using the Geneious 8.1.9 suit (Biomatters, inc).

## Additional files

Additional file 1: Supplemental figures 1-5 in a single web format file. (MHT 5228 kb)

Additional file 2: Supplemental tables 1-9 in a single file. (DOCX 90 kb)

Additional file 3: Link to supplemental spreadsheet. (DOCX 11 kb)

## Data Availability

This project was registered at the National Center for Biotechnology Information (NCBI) under the accession BioProject ID PRJNA359481. 4,715 nucleotide mRNA and protein sequences were deposited to the Transcriptome Shotgun Assembly portal of GenBank under the accession GFDG00000000. The version described in this paper is the first version, GFDG01000000. The virus sequences corresponding to *Haematobia irritans* Nora virus and *Haematobia irritans* densovirus have been deposited in GenBank under accession numbers MK643150 and MK643151, respectively.

## Abbreviations

aa: Amino acid
AI: Amino acid identity
CC: Complete coding
Denso_VP4: Capsid protein VP4
E.I.: Expression index
HEL: Helicase domain
HiDV: *Haematobia irritans* densovirus
HiNV: *Haematobia irritans* Nora virus
ICTV: International Committee on Taxonomy of Viruses
JHBP: Juvenile hormone binding proteins
LRDV: Linvill Road Virus
nt: Nucleotide
NV: Nora virus
OBP: Odorant binding proteins
OCP: Outer capsid protein
ORF: Open reading frame
Parvo_CP: Parvovirus coat protein N domain
PRO: Serine protease
RdRP: RNA dependent RNA polymerase domain
VSR: Viral silencing suppressor
