# Supplementary material for: An insight into the sialome, mialome and virome of the horn fly, Haematobia irritans
Source: BMC Genomics. 2019 Jul 29;20:616. doi: 10.1186/s12864-019-5984-7 (PMC6664567; doi:10.1186/s12864-019-5984-7)
Supplement: Supplementary file 2 — Supplemental tables 1-9 in a single file. (DOCX 90 kb) [file 12864_2019_5984_MOESM2_ESM.docx]

| Table S1: Functional classification and abundance of transcripts with RPKM > 10 from the salivary gland (SG) and midgut (MG) libraries | | | | | | |
| --- | --- | --- | --- | --- | --- | --- |
|  |  |  |  |  |  |  |
| **Class** | **Average RPKM SG** | **SE** | **Average RPKM MG** | **SE** | **SG/MG *** | **N** |
| Transcription machinery | 619.45 | 279.18 | 251.29 | 54.92 | 2.4651 | 180 |
| Protein export machinery | 96.98 | 89.52 | 44.73 | 14.01 | 2.1683 | 191 |
| Secreted | 1,213.48 | 127.09 | 634.33 | 44.96 | 1.9130 | 1,787 |
| Unknown | 303.24 | 55.17 | 227.32 | 18.50 | 1.3340 | 1,649 |
| Storage | 54.47 | 23.38 | 94.81 | 28.30 | 0.5745 | 38 |
| Nuclear regulation | 40.98 | 20.90 | 130.02 | 55.59 | 0.3152 | 57 |
| Protein synthesis machinery | 70.89 | 3.69 | 283.83 | 15.39 | 0.2498 | 464 |
| Metabolism | 50.31 | 11.20 | 246.84 | 39.63 | 0.2038 | 914 |
| Unknown, conserved | 8.55 | 1.67 | 49.91 | 5.59 | 0.1713 | 335 |
| Transcription factor | 4.41 | 0.67 | 26.81 | 2.72 | 0.1646 | 33 |
| Proteasome machinery | 9.16 | 2.83 | 56.21 | 10.77 | 0.1629 | 129 |
| Nuclear export | 1.38 | 0.27 | 11.52 | 0.95 | 0.1196 | 9 |
| Signal transduction | 5.27 | 0.72 | 53.64 | 5.28 | 0.0982 | 294 |
| Transporters/storage | 4.50 | 0.68 | 58.50 | 6.69 | 0.0770 | 227 |
| Oxidant metabolism/detoxification | 3.33 | 0.56 | 46.79 | 5.36 | 0.0712 | 118 |
| Transposable element | 3.18 | 0.59 | 45.50 | 24.31 | 0.0698 | 51 |
| Cytoskeletal | 2.15 | 0.27 | 47.11 | 5.72 | 0.0456 | 135 |
| Extracellular matrix/cell adhesion | 2.70 | 0.76 | 110.74 | 27.86 | 0.0244 | 85 |
| Protein modification machinery | 4.62 | 0.54 | 444.55 | 102.15 | 0.0104 | 333 |
| Immunity | 4.64 | 1.23 | 458.62 | 118.24 | 0.0101 | 104 |
| Viral | 0.14 | 0.04 | 303.30 | 76.93 | 0.0004 | 21 |
|  |  |  |  |  |  |  |
| Total |  |  |  |  |  | 7,154 |

* The darkest blue background color represents the lowest value, the most intense red represents the largest value, while yellow represents average values.

Table S2: Functional classification and expression levels of transcripts overexpressed in the salivary glands when compared to those from midguts.

| \| **Class** \| **Average E.I. *** \| **SE** \| **N** \| \| --- \| --- \| --- \| --- \| \| **Secreted** \|  \|  \|  \| \| **Enzymes** \|  \|  \|  \| \| DNAse \| 8.96 \| 0.64 \| 7 \| \| Serine Proteases \| 0.05 \| 0.01 \| 8 \| \| Lipases \| 0.42 \| 0.10 \| 12 \| \| **Immune related** \| 0.11 \| 0.04 \| 2 \| \| **Small molecule binding domains** \|  \|  \|  \| \| Lipocalin \| 0.02 \| 0.00 \| 2 \| \| Odorant/pheromone binding domains \| 7.69 \|  \| 1 \| \| **Antigen 5 family** \| 25.49 \| 4.00 \| 48 \| \| **Conserved secreted proteins** \| 0.05 \| 0.01 \| 9 \| \| **Stomoxyini specific** \|  \|  \|  \| \| Hematobin \| 24.04 \| 3.19 \| 28 \| \| Thrombostasin \| 18.71 \| 2.18 \| 55 \| \| 2 kDa acidic peptide \| 0.57 \| 0.01 \| 5 \| \| 2.02 kDa alkaline secreted peptide \| 0.25 \| 0.02 \| 4 \| \| 3 kDa alkaline secreted peptide \| 0.23 \| 0.12 \| 6 \| \| 3.5 kDa alkaline salivary peptide \| 45.57 \| 6.32 \| 5 \| \| 5.4 kDa acidic secreted peptide \| 0.33 \| 0.05 \| 18 \| \| 7.4 kDa salivary peptide \| 4.97 \| 1.10 \| 42 \| \| 8.5 kDa alkaline salivary peptide \| 9.89 \| 0.67 \| 4 \| \| 10.8 kDa alkaline salivary protein \| 4.55 \| 0.18 \| 4 \| \| 13.7 kDa alkaline salivary protein \| 12.66 \| 2.62 \| 2 \| \| 26.4 kDa family \| 0.07 \| 0.00 \| 2 \| \| Glycine rich family \| 0.08 \| 0.01 \| 16 \| \| **Other secreted proteins** \|  \|  \|  \| \| Mucins \| 1.71 \| 0.59 \| 11 \| \| Unknown families, secreted \| 7.26 \| 1.90 \| 47 \| \| **Putative housekeeping function** \|  \|  \|  \| \| Immunity \| 0.07 \| 0.00 \| 2 \| \| Nuclear regulation \| 0.01 \| 0.00 \| 5 \| \| Protein export \| 26.97 \|  \| 1 \| \| Protein modification \| 0.07 \|  \| 1 \| \| Protein synthesis machinery \| 0.03 \| 0.01 \| 2 \| \| Transcription machinery \| 36.58 \|  \| 1 \| \| Transporters and channels \| 0.01 \| 0.00 \| 4 \| \| Unknown conserved \| 0.23 \| 0.03 \| 8 \| \| **Transposable element** \| 0.02 \|  \| 1 \| \| **Unknown product** \| 6.17 \| 1.39 \| 61 \| |  |  |  |  |  |
| --- | --- | --- | --- | --- | --- | --- | --- | --- | --- | --- | --- | --- | --- | --- | --- | --- | --- | --- | --- | --- | --- | --- | --- | --- | --- | --- | --- | --- | --- | --- | --- | --- | --- | --- | --- | --- | --- | --- | --- | --- | --- | --- | --- | --- | --- | --- | --- | --- | --- | --- | --- | --- | --- | --- | --- | --- | --- | --- | --- | --- | --- | --- | --- | --- | --- | --- | --- | --- | --- | --- | --- | --- | --- | --- | --- | --- | --- | --- | --- | --- | --- | --- | --- | --- | --- | --- | --- | --- | --- | --- | --- | --- | --- | --- | --- | --- | --- | --- | --- | --- | --- | --- | --- | --- | --- | --- | --- | --- | --- | --- | --- | --- | --- | --- | --- | --- | --- | --- | --- | --- | --- | --- | --- | --- | --- | --- | --- | --- | --- | --- | --- | --- | --- | --- | --- | --- | --- | --- | --- | --- | --- | --- | --- | --- | --- | --- | --- | --- | --- | --- | --- | --- | --- | --- | --- | --- | --- | --- | --- | --- | --- | --- | --- | --- | --- |

* EI: Expression Index. The darkest blue background color represents the lowest value, the most intense red represents the largest value, while yellow represents average values.

Table S3: Functional classification and expression levels of transcripts overexpressed in the midgut when compared to those from salivary glands.

| **Class** | **Average E.I. *** | **SE** | **N** | **Percent of total transcripts** |
| --- | --- | --- | --- | --- |
| **Digestive enzymes** |  |  |  |  |
| **Proteases** |  |  |  |  |
| Serine proteases | 11.45 | 1.33 | 274 | 18.53 |
| Metalloproteases | 0.21 | 0.05 | 9 | 0.61 |
| Threonine peptidases | 0.49 | 0.04 | 3 | 0.20 |
| Aminopeptidase | 0.65 | 0.18 | 18 | 1.22 |
| Carboxypeptidase | 3.73 | 0.94 | 25 | 1.69 |
| Gamma-glutamyl hydrolase | 0.22 | 0.01 | 4 | 0.27 |
| Dipeptidyl peptidases | 1.20 | 0.52 | 8 | 0.54 |
| **Glycosidases** | 0.49 | 0.07 | 18 | 1.22 |
| **Short chain glycosidases** | 0.44 | 0.08 | 12 | 0.81 |
| **Nucleotidases** | 0.29 | 0.07 | 7 | 0.47 |
| **Lipases** | 0.99 | 0.15 | 37 | 2.50 |
| **Protease inhibitors** |  |  |  |  |
| Cystatin | 0.06 | 0.00 | 2 | 0.14 |
| Serpins | 0.15 | 0.01 | 11 | 0.74 |
| Kazal-type | 12.78 | 7.65 | 5 | 0.34 |
| **Peritrophic matrix** |  |  |  |  |
| Peritrophins | 0.33 | 0.16 | 19 | 1.28 |
| Mucins | 0.31 | 0.05 | 37 | 2.50 |
| **Cytoskeletal proteins** | 0.27 | 0.08 | 14 | 0.95 |
| **Transporters and channels** | 0.56 | 0.11 | 49 | 3.31 |
| **Proteins with lipid binding domains** | 3.95 | 1.04 | 29 | 1.96 |
| **Immunity-related** |  |  |  |  |
| Antimicrobial peptides | 9.50 | 2.59 | 27 | 1.83 |
| Pathogen recognition proteins and lectins | 1.19 | 0.22 | 20 | 1.35 |
| Tyrosinase inhibitor | 0.93 | 0.06 | 10 | 0.68 |
| **Secreted proteins of unknown function** | 7.35 | 0.84 | 369 | 24.95 |
| **Detoxification** | 0.15 | 0.01 | 12 | 0.81 |
| Oxidant metabolism/Detoxification | 0.39 | 0.05 | 13 | 0.88 |
| **Metabolism** |  |  |  |  |
| Energy metabolism | 0.50 | 0.29 | 9 | 0.61 |
| Amino acid metabolism | 0.09 | 0.02 | 3 | 0.20 |
| Carbohydrate metabolism | 0.21 | 0.00 | 3 | 0.20 |
| Intermediary metabolism | 5.01 | 4.82 | 2 | 0.14 |
| Lipid metabolism | 1.57 | 0.42 | 50 | 3.38 |
| Nucleotide metabolism | 0.20 | 0.05 | 7 | 0.47 |
| **Transcription and translation** |  |  |  |  |
| Transcription machinery | 6.15 | 0.71 | 38 | 2.57 |
| Protein synthesis machinery | 0.21 | 0.02 | 6 | 0.41 |
| Protein modification | 0.21 | 0.10 | 19 | 1.28 |
| Proteasome machinery | 0.28 | 0.08 | 2 | 0.14 |
| **Signal transduction** |  |  |  | 0.00 |
| Neuropeptides | 0.12 | 0.00 | 4 | 0.27 |
| Other signal transduction-related transcripts | 0.58 | 0.15 | 45 | 3.04 |
| **Unknown conserved** | 0.47 | 0.15 | 6 | 0.41 |
| **Unknown conserved membrane protein** | 0.18 | 0.04 | 11 | 0.74 |
| **Unknown product** | 2.38 | 0.47 | 220 | 14.87 |
| **Viral products** |  |  |  |  |
| Nora virus | 0.41 | 0.11 | 11 | 0.74 |
| Filoviridae | 0.50 | 0.02 | 3 | 0.20 |
| Densovirus | 4.96 | 0.92 | 8 | 0.54 |

* EI: Expression Index. The darkest blue background color represents the lowest value, the most intense red represents the largest value, while yellow represents average values.

Table S4: Classification based on the Merops database and expression levels of transcripts coding for serine endopeptidases overexpressed in the midgut when compared to those from salivary glands.

| **Merops Clan** | **Average E.I. *** | **SE** | **N** |
| --- | --- | --- | --- |
| Trypsin alpha a | 40.45 | 8.42 | 21 |
| Trypsin alpha b | 11.25 | 1.71 | **3** |
| Trypsin zeta a | 0.07 | 0.00 | 5 |
| Trypsin zeta b | 0.56 | 0.01 | 4 |
| Trypsin zeta c | 0.23 | 0.00 | 3 |
| Trypsin zeta d | 0.98 | 0.06 | 2 |
| Trypsin zeta e | 0.47 | 0.01 | 2 |
| Trypsin zeta f | 0.69 | 0.19 | 2 |
| Try29Fa | 0.22 | 0.01 | 3 |
| Try29Fb | 26.52 | 15.32 | 5 |
| Try29Fc | 55.97 | 18.21 | 2 |
| Try29Fd | 6.80 | 0.19 | 2 |
| Try29Fe | 7.28 | 0.07 | 2 |
| Try29Ff | 60.95 | 13.93 | 3 |
| Trypsin lambda | 4.84 | 0.24 | 2 |
| CG17571A | 4.35 | 1.52 | 18 |
| CG17571 | 0.60 | 0.11 | 4 |
| CG9676a | 6.19 | 1.81 | 12 |
| CG9676b | 8.73 | 0.77 | 5 |
| CG9676c | 3.82 | 0.21 | 4 |
| CG9676d | 3.52 | 0.72 | 4 |
| CG9676e | 0.07 | 0.00 | 3 |
| CG9676f | 0.11 | 0.00 | 3 |
| CG9676g | 1.77 | 0.20 | 2 |
| CG9676h | 9.23 | 0.18 | 2 |
| CG9676i | 3.43 | 0.28 | 2 |
| CG7542a | 72.07 | 7.44 | 7 |
| CG7542b | 10.42 | 4.80 | 2 |
| CG6048a | 4.64 | 0.17 | 7 |
| CG6048b | 3.87 | 0.37 | 5 |
| CG6041a | 0.53 | 0.01 | 6 |
| CG6041b | 0.67 | 0.02 | 3 |
| CG5246a | 0.78 | 0.02 | 4 |
| CG5246b | 0.38 | 0.02 | 3 |
| CG5246c | 1.56 | 0.19 | 2 |
| CG18493 | 2.35 | 0.23 | 4 |
| CG3734 | 0.96 | 0.11 | 4 |
| CG5233 | 0.79 | 0.31 | 5 |
| CG11864 | 1.59 | 0.01 | 2 |
| CG8299 | 1.03 | 0.00 | 2 |
| CG7142 | 0.09 | 0.00 | 2 |
| CG14780 | 0.17 | 0.05 | 2 |
| jonah | 24.78 | 11.65 | 6 |
| jonah 65Aiv a | 8.08 | 7.98 | 5 |
| jonah 65Aiv b | 0.99 | 0.02 | 2 |
| jonah 65Aiv c | 12.02 | 4.12 | 2 |
| jonah 65Aiv d | 0.10 | 0.00 | 2 |
| Lectizyme 1 | 13.60 | 5.94 | 17 |
| Lectizyme 2 | 16.67 | 4.16 | 5 |
| Serine protease with leucine zipper | 0.09 | 0.01 | 6 |
| Uncharacterized a | 7.44 | 0.51 | 7 |
| Uncharacterized b | 9.38 | 0.62 | 6 |
| Uncharacterized c | 0.33 | 0.00 | 2 |
| Uncharacterized d | 0.22 | 0.01 | 2 |
| Uncharacterized e | 2.89 | 0.73 | 2 |
| Uncharacterized f | 3.44 | 0.12 | 2 |
| Uncharacterized g | 18.62 | 7.43 | 5 |
| Uncharacterized h | 2.92 | 0.17 | 3 |
| Other serine proteases | 7.83 | 3.47 | 20 |

* EI: Expression Index. The darkest blue background color represents the lowest value, the most intense red represents the largest value, while yellow represents average values.

Table S5: Classification of transcripts coding for secreted peptides of unknown function that are overexpressed at least 16-fold in the midgut of *Haematobia irritans* when compared to expression in the salivary gland library.

| **Transcript group** | **Average E.I. *** | **SE** | **N** |
| --- | --- | --- | --- |
| Highly expressed midgut secreted peptide of the 5.7 kDa family | 81.49 | 8.62 | 5 |
| Highly expressed conserved 9.1 kDa midgut peptide | 68.87 | 1.18 | 3 |
| Highly expressed 10.5 kDa midgut secreted peptide | 59.22 | 14.82 | 2 |
| Highly expressed unique midgut secreted peptide of the 6.71 kDa family | 57.73 | 13.30 | 5 |
| Highly expressed unique 7 kDa midgut peptide | 26.10 | 4.01 | 17 |
| Other highly expressed midgut secreted peptides | 13.78 | 1.86 | 14 |
| Unique 5.88 kDa midgut peptide | 10.74 | 3.84 | 4 |
| Conserved 34.1 kDa midgut protein | 9.20 | 2.86 | 9 |
| Conserved 12.5 kDa midgut protein | 6.90 | 1.32 | 11 |
| Conserved 10.5 kDa midgut peptide | 4.73 | 0.93 | 10 |
| Probable peptidase fragment | 4.47 | 2.59 | 12 |
| Unique 7.67 kDa peptide | 4.04 | 0.34 | 7 |
| Conserved 25.7 kDa midgut protein | 3.76 | 0.72 | 16 |
| Conserved 10.9 kDa midgut peptide | 3.01 | 0.59 | 13 |
| Conserved 10.8 kDa midgut protein | 2.15 | 0.85 | 7 |
| Other midgut secreted peptides | 0.76 | 0.07 | 188 |
| Unique 14.3 kDa WWWW midgut peptide | 0.69 | 0.05 | 2 |
| Conserved 25.7 kDa midgut protein | 0.27 | 0.01 | 6 |
| Conserved 14.3 kDa midgut protein | 0.12 | 0.01 | 5 |
| Unique 11.9 kDa midgut peptide | 0.10 | 0.00 | 4 |
| Unique 8.21 kDa midgut peptide | 0.08 | 0.00 | 4 |
| Conserved 9.28 kDa midgut peptide | 0.07 | 0.01 | 5 |
| Unique 7.13 kDa peptide | 0.07 | 0.00 | 6 |

* EI: Expression Index. The darkest blue background color represents the lowest value, the most intense red represents the largest value, while yellow represents average values.

Table S6: Classification of transcripts coding for channels and transporters that are overexpressed at least 16-fold in the midgut of *Haematobia irritans* when compared to expression in the salivary gland library.

| **Transcript class** | **Average E.I. *** | **SE** | **N** |
| --- | --- | --- | --- |
| Amino acid transporter | 0.24 | 0.14 | 4 |
| Bumetanide-sensitive Na+(K+)-chloride cotransporter | 0.10 | 0.00 | 7 |
| Copper transporter | 0.07 | 0.00 | 2 |
| Inorganic phosphate cotransporter | 0.63 | 0.24 | 5 |
| K+/Cl- cotransporter KCC1 | 0.10 | 0.00 | 1 |
| Monocarboxylate transporter | 0.13 | 0.01 | 4 |
| Peptide transporter | 1.58 | 0.09 | 2 |
| Permease of the major facilitator superfamily | 0.49 | 0.14 | 7 |
| Probable glucose transporter | 0.12 | 0.00 | 4 |
| Sodium-dependent multivitamin transporter | 0.07 | 0.00 | 3 |
| Vacuolar H+-ATPase V0 sector subunit a | 0.44 | 0.03 | 4 |
| Carbonic anhydrase | 2.12 | 0.36 | 6 |

* EI: Expression Index. The darkest blue background color represents the lowest value, the most intense red represents the largest value, while yellow represents average values.

Table S7: Virus derived reads determined by mapping using Bowtie2 with standard parameters. Values were calculated in our dataset (BioProject PRJNA359481), and additional publicly available studies of High-throughput sequencing of *H. irritans*. Read per million (RPM) are in bold and heatmap values adjusted from white to red (highest value) for each BioProject.

| **Run** | **BioProject** | **BioSample** | **Sample name** | **Total reads** | **HiNV reads** | **HiNV RPM** | **HiDV reads** | **HiDV RPM** |
| --- | --- | --- | --- | --- | --- | --- | --- | --- |
| [SRR5136555](https://www.ncbi.nlm.nih.gov/Traces/sra/?run=SRR5136555) | [PRJNA359481](https://www.ncbi.nlm.nih.gov/bioproject/PRJNA359481) | [SAMN06192182](https://www.ncbi.nlm.nih.gov/biosample/SAMN06192182) | HI-MG | 37442518 | 206662 | **5519** | 476886 | **12736** |
| [SRR5136554](https://www.ncbi.nlm.nih.gov/Traces/sra/?run=SRR5136554) | [PRJNA359481](https://www.ncbi.nlm.nih.gov/bioproject/PRJNA359481) | [SAMN06192182](https://www.ncbi.nlm.nih.gov/biosample/SAMN06192182) | HI-MG | 36370476 | 248211 | **6825** | 772352 | **21236** |
| [SRR5136553](https://www.ncbi.nlm.nih.gov/Traces/sra/?run=SRR5136553) | [PRJNA359481](https://www.ncbi.nlm.nih.gov/bioproject/PRJNA359481) | [SAMN06192181](https://www.ncbi.nlm.nih.gov/biosample/SAMN06192181) | Hi-SG | 38999009 | 81 | **2** | 43 | **1** |
| [SRR5136552](https://www.ncbi.nlm.nih.gov/Traces/sra/?run=SRR5136552) | [PRJNA359481](https://www.ncbi.nlm.nih.gov/bioproject/PRJNA359481) | [SAMN06192181](https://www.ncbi.nlm.nih.gov/biosample/SAMN06192181) | Hi-SG | 32971890 | 694 | **21** | 241 | **7** |
| [SRR6659629](https://www.ncbi.nlm.nih.gov/Traces/sra/?run=SRR6659629) | [PRJNA429442](https://www.ncbi.nlm.nih.gov/bioproject/PRJNA429442) | [SAMN08355025](https://www.ncbi.nlm.nih.gov/biosample/SAMN08355025) | PT Surviving Males | 68856572 | 528429 | **7674** | 0 | **0** |
| [SRR6659628](https://www.ncbi.nlm.nih.gov/Traces/sra/?run=SRR6659628) | [PRJNA429442](https://www.ncbi.nlm.nih.gov/bioproject/PRJNA429442) | [SAMN08355026](https://www.ncbi.nlm.nih.gov/biosample/SAMN08355026) | PPBT Killed Males | 65427160 | 1855 | **28** | 0 | **0** |
| [SRR6659627](https://www.ncbi.nlm.nih.gov/Traces/sra/?run=SRR6659627) | [PRJNA429442](https://www.ncbi.nlm.nih.gov/bioproject/PRJNA429442) | [SAMN08355024](https://www.ncbi.nlm.nih.gov/biosample/SAMN08355024) | Males | 72324378 | 0 | **0** | 4941 | **68** |
| [SRR6659626](https://www.ncbi.nlm.nih.gov/Traces/sra/?run=SRR6659626) | [PRJNA429442](https://www.ncbi.nlm.nih.gov/bioproject/PRJNA429442) | [SAMN08355024](https://www.ncbi.nlm.nih.gov/biosample/SAMN08355024) | Males | 60050116 | 0 | **0** | 24543 | **409** |
| [SRR6659625](https://www.ncbi.nlm.nih.gov/Traces/sra/?run=SRR6659625) | [PRJNA429442](https://www.ncbi.nlm.nih.gov/bioproject/PRJNA429442) | [SAMN08355023](https://www.ncbi.nlm.nih.gov/biosample/SAMN08355023) | Females | 62225974 | 0 | **0** | 0 | **0** |
| [SRR6659624](https://www.ncbi.nlm.nih.gov/Traces/sra/?run=SRR6659624) | [PRJNA429442](https://www.ncbi.nlm.nih.gov/bioproject/PRJNA429442) | [SAMN08355023](https://www.ncbi.nlm.nih.gov/biosample/SAMN08355023) | Females | 72445844 | 0 | **0** | 19 | **0** |
| [SRR6231671](https://www.ncbi.nlm.nih.gov/Traces/sra/?run=SRR6231671) | [PRJNA30967](https://www.ncbi.nlm.nih.gov/bioproject/PRJNA30967) | [SAMN07830370](https://www.ncbi.nlm.nih.gov/biosample/SAMN07830370) | hornfly_adult24hrfeed | 47070858 | 0 | **0** | 0 | **0** |
| [SRR6231670](https://www.ncbi.nlm.nih.gov/Traces/sra/?run=SRR6231670) | [PRJNA30967](https://www.ncbi.nlm.nih.gov/bioproject/PRJNA30967) | [SAMN07830369](https://www.ncbi.nlm.nih.gov/biosample/SAMN07830369) | hornfly_adult4hrfeed | 50512062 | 0 | **0** | 2 | **0** |
| [SRR6231669](https://www.ncbi.nlm.nih.gov/Traces/sra/?run=SRR6231669) | [PRJNA30967](https://www.ncbi.nlm.nih.gov/bioproject/PRJNA30967) | [SAMN07830372](https://www.ncbi.nlm.nih.gov/biosample/SAMN07830372) | hornfly_pupae3d | 51859710 | 0 | **0** | 1322 | **25** |
| [SRR6231668](https://www.ncbi.nlm.nih.gov/Traces/sra/?run=SRR6231668) | [PRJNA30967](https://www.ncbi.nlm.nih.gov/bioproject/PRJNA30967) | [SAMN07830371](https://www.ncbi.nlm.nih.gov/biosample/SAMN07830371) | hornfly_pupae1d | 53975828 | 175 | **3** | 102 | **2** |
| [SRR6231667](https://www.ncbi.nlm.nih.gov/Traces/sra/?run=SRR6231667) | [PRJNA30967](https://www.ncbi.nlm.nih.gov/bioproject/PRJNA30967) | [SAMN07830366](https://www.ncbi.nlm.nih.gov/biosample/SAMN07830366) | hornfly_testes | 89085268 | 0 | **0** | 0 | **0** |
| [SRR6231666](https://www.ncbi.nlm.nih.gov/Traces/sra/?run=SRR6231666) | [PRJNA30967](https://www.ncbi.nlm.nih.gov/bioproject/PRJNA30967) | [SAMN07830365](https://www.ncbi.nlm.nih.gov/biosample/SAMN07830365) | hornfly_SalGland | 27461942 | 0 | **0** | 0 | **0** |
| [SRR6231665](https://www.ncbi.nlm.nih.gov/Traces/sra/?run=SRR6231665) | [PRJNA30967](https://www.ncbi.nlm.nih.gov/bioproject/PRJNA30967) | [SAMN07830368](https://www.ncbi.nlm.nih.gov/biosample/SAMN07830368) | hornfly_adult2hrfeed | 51829048 | 0 | **0** | 3181 | **61** |
| [SRR6231664](https://www.ncbi.nlm.nih.gov/Traces/sra/?run=SRR6231664) | [PRJNA30967](https://www.ncbi.nlm.nih.gov/bioproject/PRJNA30967) | [SAMN07830367](https://www.ncbi.nlm.nih.gov/biosample/SAMN07830367) | hornfly_adult0hrfeed | 44820832 | 0 | **0** | 1 | **0** |
| [SRR6231663](https://www.ncbi.nlm.nih.gov/Traces/sra/?run=SRR6231663) | [PRJNA30967](https://www.ncbi.nlm.nih.gov/bioproject/PRJNA30967) | [SAMN07830364](https://www.ncbi.nlm.nih.gov/biosample/SAMN07830364) | hornfly_ovary | 39372642 | 0 | **0** | 0 | **0** |
| [SRR6231662](https://www.ncbi.nlm.nih.gov/Traces/sra/?run=SRR6231662) | [PRJNA30967](https://www.ncbi.nlm.nih.gov/bioproject/PRJNA30967) | [SAMN07830363](https://www.ncbi.nlm.nih.gov/biosample/SAMN07830363) | hornfly_MalpighianT | 34421850 | 0 | **0** | 0 | **0** |
| [SRR6231661](https://www.ncbi.nlm.nih.gov/Traces/sra/?run=SRR6231661) | [PRJNA30967](https://www.ncbi.nlm.nih.gov/bioproject/PRJNA30967) | [SAMN07830360](https://www.ncbi.nlm.nih.gov/biosample/SAMN07830360) | hornfly_egg9hr | 33832868 | 0 | **0** | 0 | **0** |
| [SRR6231660](https://www.ncbi.nlm.nih.gov/Traces/sra/?run=SRR6231660) | [PRJNA30967](https://www.ncbi.nlm.nih.gov/bioproject/PRJNA30967) | [SAMN07830359](https://www.ncbi.nlm.nih.gov/biosample/SAMN07830359) | hornfly_egg4hr | 31907062 | 0 | **0** | 0 | **0** |
| [SRR6231659](https://www.ncbi.nlm.nih.gov/Traces/sra/?run=SRR6231659) | [PRJNA30967](https://www.ncbi.nlm.nih.gov/bioproject/PRJNA30967) | [SAMN07830362](https://www.ncbi.nlm.nih.gov/biosample/SAMN07830362) | hornfly_legs | 38066002 | 0 | **0** | 0 | **0** |
| [SRR6231658](https://www.ncbi.nlm.nih.gov/Traces/sra/?run=SRR6231658) | [PRJNA30967](https://www.ncbi.nlm.nih.gov/bioproject/PRJNA30967) | [SAMN07830361](https://www.ncbi.nlm.nih.gov/biosample/SAMN07830361) | hornfly_gut | 33327082 | 0 | **0** | 0 | **0** |
| [SRR6231655](https://www.ncbi.nlm.nih.gov/Traces/sra/?run=SRR6231655) | [PRJNA30967](https://www.ncbi.nlm.nih.gov/bioproject/PRJNA30967) | [SAMN07830358](https://www.ncbi.nlm.nih.gov/biosample/SAMN07830358) | hornfly_egg2hr | 38158656 | 0 | **0** | 0 | **0** |
| [SRR6231654](https://www.ncbi.nlm.nih.gov/Traces/sra/?run=SRR6231654) | [PRJNA30967](https://www.ncbi.nlm.nih.gov/bioproject/PRJNA30967) | [SAMN07830357](https://www.ncbi.nlm.nih.gov/biosample/SAMN07830357) | hornfly_egg0hr | 34417392 | 0 | **0** | 0 | **0** |
| [SRR003192](https://www.ncbi.nlm.nih.gov/Traces/sra/?run=SRR003192) | [PRJNA30967](https://www.ncbi.nlm.nih.gov/bioproject/PRJNA30967) | [SAMN00000589](https://www.ncbi.nlm.nih.gov/biosample/SAMN00000589) | horn fly adult male | 71550 | 0 | **0** | 0 | **0** |
| [SRR003191](https://www.ncbi.nlm.nih.gov/Traces/sra/?run=SRR003191) | [PRJNA30967](https://www.ncbi.nlm.nih.gov/bioproject/PRJNA30967) | [SAMN00000588](https://www.ncbi.nlm.nih.gov/biosample/SAMN00000588) | Horn fly adult female | 85769 | 0 | **0** | 5 | **58** |
| [SRR003190](https://www.ncbi.nlm.nih.gov/Traces/sra/?run=SRR003190) | [PRJNA30967](https://www.ncbi.nlm.nih.gov/bioproject/PRJNA30967) | [SAMN00000586](https://www.ncbi.nlm.nih.gov/biosample/SAMN00000586) | Horn fly egg | 73512 | 0 | **0** | 0 | **0** |
| [SRR003189](https://www.ncbi.nlm.nih.gov/Traces/sra/?run=SRR003189) | [PRJNA30967](https://www.ncbi.nlm.nih.gov/bioproject/PRJNA30967) | [SAMN00000587](https://www.ncbi.nlm.nih.gov/biosample/SAMN00000587) | Horn fly larvae | 100603 | 0 | **0** | 0 | **0** |

Table S8: Variable sites of HiDV based on virus RNA reads detected in SRR6659626 and SRR6659627 (*H. irritans* whole adults from Saint Gabriel, LA, USA). Polymorphism are indicated in relation with the consensus sequence of HiDV-URU strain determined for BioProject PRJNA359481. Only variable sites with a frequency >0.25 of virus reads are shown.

| **Variant** | **Location** | **CDS position** | **Amino acid change** | **Position within codon** | **Change** | **Codon change** | **Polymorphism type** | **Protein effect** | **Variant frequency** | **Variant P-Value** |
| --- | --- | --- | --- | --- | --- | --- | --- | --- | --- | --- |
| Var1 | 104 | 23 | S -> N | 2 | G -> A | AGT -> AAT | SNP (transition) | Substitution | 100.0% | 1.0E-92 |
| Var2 | 110 | 29 | T -> R | 2 | C -> G | ACA -> AGA | SNP (transversion) | Substitution | 87.3% | 1.9E-88 |
| Var3 | 113 | 32 | N -> S | 2 | A -> G | AAT -> AGT | SNP (transition) | Substitution | 100.0% | 1.0E-134 |
| Var4 | 145 | 9 |  | 3 | G -> A | GAG -> GAA | SNP (transition) | None | 100.0% | 1.0E-322 |
| Var5 | 460 | 324 |  | 3 | C -> T | ACC -> ACT | SNP (transition) | None | 52.8% | 1.4E-96 |
| Var6 | 462 | 381 |  | 3 | G -> C | CTG -> CTC | SNP (transversion) | None | 49.6% | 1.5E-95 |
| Var7 | 495 | 414 |  | 3 | G -> A | AAG -> AAA | SNP (transition) | None | 46.2% | 1.6E-99 |
| Var8 | 598 | 517 |  | 1 | C -> A | CGA -> AGA | SNP (transversion) | None | 66.9% | 7.6E-134 |
| Var9 | 898 | 762 |  | 3 | G -> A | AAG -> AAA | SNP (transition) | None | 98.3% | 6.4E-223 |
| Var10 | 1,068 | 987 |  | 3 | T -> C | GTT -> GTC | SNP (transition) | None | 100.0% | 1.0E-292 |
| Var11 | 1,074 | 993 |  | 3 | C -> T | TTC -> TTT | SNP (transition) | None | 88.5% | 7.4E-199 |
| Var12 | 1,191 | 1110 |  | 3 | A -> G | CAA -> CAG | SNP (transition) | None | 75.3% | 4.2E-99 |
| Var13 | 1,194 | 1113 |  | 3 | C -> T | TTC -> TTT | SNP (transition) | None | 79.3% | 7.9E-81 |
| Var14 | 1,29 | 1209 | D -> E | 3 | T -> A | GAT -> GAA | SNP (transversion) | Substitution | 100.0% | 1.0E-268 |
| Var15 | 1,35 | 1269 |  | 3 | C -> T | AAC -> AAT | SNP (transition) | None | 98.8% | 1.6E-326 |
| Var16 | 1,452 | 1371 |  | 3 | T -> C | GAT -> GAC | SNP (transition) | None | 99.5% | 2.0E-404 |
| Var17 | 1,509 | 1428 |  | 3 | G -> T | CTG -> CTT | SNP (transversion) | None | 75.6% | 1.2E-257 |
| Var18 | 1,521 | 1440 |  | 3 | T -> C | GGT -> GGC | SNP (transition) | None | 100.0% | 1.0E-300 |
| Var19 | 1,656 | 1575 |  | 3 | T -> A | CCT -> CCA | SNP (transversion) | None | 100.0% | 1.0E-238 |
| Var20 | 1,665 | 1584 |  | 3 | T -> A | GTT -> GTA | SNP (transversion) | None | 100.0% | 1.0E-164 |
| Var21 | 1,674 | 1593 |  | 3 | C -> T | AAC -> AAT | SNP (transition) | None | 100.0% | 1.0E-136 |
| Var22 | 1,737 | 1656 |  | 3 | T -> G | GCT -> GCG | SNP (transversion) | None | 99.2% | 1.2E-234 |
| Var23 | 1,785 | 1704 |  | 3 | A -> T | TCA -> TCT | SNP (transversion) | None | 100.0% | 1.0E-12 |
| Var24 | 1,803 | 1722 |  | 3 | T -> A | ACT -> ACA | SNP (transversion) | None | 100.0% | 1.0E-152 |
| Var25 | 1,866 | 1785 |  | 3 | A -> G | GAA -> GAG | SNP (transition) | None | 100.0% | 1.0E-240 |
| Var26 | 1,875 | 1794 |  | 3 | A -> G | CTA -> CTG | SNP (transition) | None | 100.0% | 1.0E-270 |
| Var27 | 1,953 | 1872 |  | 3 | T -> A | ACT -> ACA | SNP (transversion) | None | 100.0% | 1.0E-120 |
| Var28 | 1,959 | 1878 |  | 3 | T -> C | AAT -> AAC | SNP (transition) | None | 100.0% | 1.0E-94 |
| Var29 | 2,101 | 63 |  | 3 | C -> T | GGC -> GGT | SNP (transition) | None | 83.0% | 3.1E-303 |
| Var30 | 2,167 | 129 |  | 3 | C -> T | ACC -> ACT | SNP (transition) | None | 92.4% | 6.5E-598 |
| Var31 | 2,173 | 135 |  | 3 | G -> A | GAG -> GAA | SNP (transition) | None | 100.0% | 1.0E-664 |
| Var32 | 2,185 | 147 |  | 3 | A -> G | AAA -> AAG | SNP (transition) | None | 92.0% | 4.2E-581 |
| Var33 | 2,365 | 327 | S -> SHES | 3 | +ACACGAGTC | TCT -> TCA,CAC,GAG,TCT | Insertion | Insertion | 65.9% | 2.6E-188 |
| Var34 | 2,386 | 348 |  | 3 | G -> A | CCG -> CCA | SNP (transition) | None | 100.0% | 2.5E-768 |
| Var35 | 2,413 | 375 |  | 3 | G -> T | CCG -> CCT | SNP (transversion) | None | 100.0% | 1.0E-848 |
| Var36 | 2,555 | 517 | S -> A | 1 | T -> G | TCA -> GCA | SNP (transversion) | Substitution | 99.6% | 1.3E-1037 |
| Var37 | 2,56 | 522 |  | 3 | C -> A | TCC -> TCA | SNP (transversion) | None | 98.5% | 1.3E-1021 |
| Var38 | 2,597 | 559 |  | 1 | C -> A | CGA -> AGA | SNP (transversion) | None | 99.8% | 1.0E-1038 |
| Var39 | 2,723 | 297 |  | 3 | G -> C | ACG -> ACC | SNP (transversion) | None | 99.8% | 5.8E-1160 |
| Var40 | 2,728 | 302 | R -> Q | 2 | GC -> AG | CGC -> CAG | Substitution | Substitution | 99.8% | 5.0E-1002 |
| Var41 | 2,739 | 701 | G -> A | 2 | G -> C | GGA -> GCA | SNP (transversion) | Substitution | 99.6% | 1.1E-953 |
| Var42 | 2,857 | 819 |  | 3 | T -> C | TAT -> TAC | SNP (transition) | None | 100.0% | 1.0E-816 |
| Var43 | 3,085 | 1047 |  | 3 | G -> A | AAG -> AAA | SNP (transition) | None | 100.0% | 1.0E-906 |
| Var44 | 3,139 | 1101 |  | 3 | G -> C | GTG -> GTC | SNP (transversion) | None | 99.7% | 3.7E-746 |
| Var45 | 3,228 | 1190 | S -> T | 2 | G -> C | AGT -> ACT | SNP (transversion) | Substitution | 100.0% | 1.0E-1042 |
| Var46 | 3,349 | 1311 |  | 3 | G -> A | ACG -> ACA | SNP (transition) | None | 99.2% | 7.7E-1122 |
| Var47 | 3,358 | 1320 |  | 3 | G -> A | TTG -> TTA | SNP (transition) | None | 99.3% | 4.0E-1118 |
| Var48 | 3,382 | 1344 |  | 3 | T -> G | GGT -> GGG | SNP (transversion) | None | 100.0% | 1.0E-804 |
| Var49 | 3,428 | 1390 | V -> T | 1 | GT -> AC | GTA -> ACA | Substitution | Substitution | 81.5% -> 82.6% | 4.4E-612 |
| Var50 | 3,436 | 1398 |  | 3 | A -> T | CCA -> CCT | SNP (transversion) | None | 76.5% | 9.8E-608 |
| Var51 | 3,448 | 1410 |  | 3 | A -> G | TCA -> TCG | SNP (transition) | None | 79.1% | 6.9E-771 |
| Var52 | 3,479 | 1441 | S -> A | 1 | T -> G | TCT -> GCT | SNP (transversion) | Substitution | 100.0% | 1.0E-960 |
| Var53 | 3,492 | 1454 | V -> A | 2 | T -> C | GTA -> GCA | SNP (transition) | Substitution | 100.0% | 1.0E-598 |
| Var54 | 3,499 | 1461 |  | 3 | T -> A | GGT -> GGA | SNP (transversion) | None | 98.8% | 2.4E-484 |
| Var55 | 3,503 | 1465 | N -> Y | 1 | A -> T | AAT -> TAT | SNP (transversion) | Substitution | 100.0% | 1.0E-500 |
| Var56 | 3,52 | 1482 |  | 3 | A -> G | ACA -> ACG | SNP (transition) | None | 43.9% | 1.0E-215 |
| Var57 | 3,541 | 1503 | EI -> DV | 3 | AA -> TG | GAA,ATA -> GAT,GTA | Substitution | Substitution | 100.0% | 1.0E-1210 |
| Var58 | 3,547 | 1509 |  | 3 | T -> C | CCT -> CCC | SNP (transition) | None | 28.1% | 1.2E-194 |
| Var59 | 3,611 | 1573 | L -> I | 1 | T -> A | TTA -> ATA | SNP (transversion) | Substitution | 81.6% | 1.2E-534 |
| Var60 | 3,706 | 1668 |  | 3 | G -> A | GTG -> GTA | SNP (transition) | None | 99.7% | 2.0E-1267 |
| Var61 | 3,787 | 1749 | M -> I | 3 | G -> T | ATG -> ATT | SNP (transversion) | Substitution | 100.0% | 1.0E-870 |
| Var62 | 3,824 | 1786 | S -> D | 1 | AG -> GA | AGT -> GAT | Substitution | Substitution | 99.4% | 2.1E-1030 |
| Var63 | 3,827 | 1789 |  | 1 | T -> C | TTA -> CTA | SNP (transition) | None | 99.3% | 5.3E-1191 |
| Var64 | 3,833 | 1795 | S -> P | 1 | T -> C | TCT -> CCT | SNP (transition) | Substitution | 99.5% | 3.9E-1233 |
| Var65 | 3,851 | 1813 | S -> T | 1 | T -> A | TCA -> ACA | SNP (transversion) | Substitution | 99.7% | 3.0E-1559 |

**Table S9**: GenBank accession numbers and assigned taxonomy of the virus sequences used for phylogenetic insights of HiDV.

| **Virus** | **GenBank acc #** | **Virus taxonomy** |
| --- | --- | --- |
| Ambidensovirus CaaDV1 | ARI46481 | Parvoviridae; Densovirinae; Ambidensovirus; unclassified Densovirus |
| Ambidensovirus CaaDV2 | ARI46485 | Parvoviridae; Densovirinae; Ambidensovirus; unclassified Densovirus |
| Blattella germanica densovirus 1 | NP_874381 | Parvoviridae; Densovirinae; Ambidensovirus |
| Bombus-associated virus Den2 | QAY29280 | Parvoviridae; Densovirinae; unclassified Densovirinae |
| Bombus cryptarum densovirus | YP_009552708 | Parvoviridae; Densovirinae; unclassified Densovirinae |
| Bombyx mori densovirus 1 | NP_542609 | Parvoviridae; Densovirinae; Iteradensovirus |
| Casphalia extranea densovirus | NP_694838 | Parvoviridae; Densovirinae; Iteradensovirus |
| Cherax quadricarinatus densovirus | YP_009134732 | Parvoviridae; Densovirinae; Ambidensovirus |
| Culex densovirus | AXQ04855 | Parvoviridae; Densovirinae; unclassified Densovirinae |
| Culex pipiens densovirus | YP_002887625 | Parvoviridae; Densovirinae; Ambidensovirus |
| Danaus plexippus plexippus iteravirus | YP_009021036 | Parvoviridae; Densovirinae; Iteradensovirus; unclassified Iteravirus |
| Dendrolimus punctatus densovirus | YP_164339 | Parvoviridae; Densovirinae; Iteradensovirus |
| Diaphorina citri densovirus | YP_009256211 | Parvoviridae; Densovirinae; Ambidensovirus; unclassified Densovirus |
| Diatraea saccharalis densovirus | NP_046813 | Parvoviridae; Densovirinae; Ambidensovirus; unclassified Densovirus |
| Dysaphis plantaginea densovirus | YP_009362129 | Parvoviridae; Densovirinae; Ambidensovirus |
| Galleria mellonella densovirus | NP_899650 | Parvoviridae; Densovirinae; Ambidensovirus |
| Helicoverpa armigera densovirus | YP_004678720 | Parvoviridae; Densovirinae; Iteradensovirus |
| Hordeum marinum Itera-like densovirus | AIT71975 | Parvoviridae; Densovirinae; Iteradensovirus; unclassified Iteravirus |
| Human CSF-associated densovirus | ANG55949 | Parvoviridae; Densovirinae; unclassified Densovirinae |
| Junonia coenia densovirus | NP_694824 | Parvoviridae; Densovirinae; Ambidensovirus |
| Linvill Road virus | AQN78650 | Parvoviridae; Densovirinae; unclassified Densovirinae |
| Lone star tick densovirus 1 | ASU47551 | Parvoviridae; Densovirinae; unclassified Densovirinae |
| Lupine feces-associated densovirus | ASM93491 | Parvoviridae; Densovirinae; Ambidensovirus; unclassified Densovirus |
| Lupine feces-associated densovirus 2 | ASM93489 | Parvoviridae; Densovirinae; Ambidensovirus; unclassified Densovirus |
| Mythimna loreyi densovirus | NP_958099 | Parvoviridae; Densovirinae; Ambidensovirus |
| Myzus persicae densovirus | NP_874376 | Parvoviridae; Densovirinae; Ambidensovirus |
| Papilio polyxenes densovirus | YP_006589928 | Parvoviridae; Densovirinae; Iteradensovirus |
| Parus major densovirus | YP_009310053 | Parvoviridae; Densovirinae; unclassified Densovirinae |
| Penaeus stylirostris penstyldensovirus 1 | NC_039043 | Parvoviridae; Densovirinae; Penstyldensovirus |
| Periplaneta fuliginosa densovirus | NP_051020 | Parvoviridae; Densovirinae; Ambidensovirus |
| Planococcus citri densovirus | ARV85890 | Parvoviridae; Densovirinae; Ambidensovirus |
| Pseudoplusia includens densovirus | YP_007003823 | Parvoviridae; Densovirinae; Ambidensovirus |
| Sea star-associated densovirus | YP_009507340 | Parvoviridae; Densovirinae; Ambidensovirus |
| Sibine fusca densovirus | YP_006576512 | Parvoviridae; Densovirinae; Iteradensovirus |
| Sitobion miscanthi densovirus | AWS20449 | Parvoviridae; Densovirinae; unclassified Densovirinae |
| Solenopsis invicta densovirus | YP_008766862 | Parvoviridae; Densovirinae; Ambidensovirus |
| Viltain virus | AQN78648 | Parvoviridae; Densovirinae; unclassified Densovirinae |
| Acheta domestica mini ambidensovirus | NC_022564 | Parvoviridae; Densovirinae |
| Aedes albopictus densovirus 2 | NC_004285 | Parvoviridae; Densovirinae; Brevidensovirus |
| Anopheles gambiae densovirus | NC_011317 | Parvoviridae; Densovirinae; Brevidensovirus |
| Decapod penstyldensovirus 1 | NC_002190 | Parvoviridae; Densovirinae; Penstyldensovirus |
| Fenneropenaeus chinensis hepandensovirus | NC_014357 | Parvoviridae; Densovirinae; Hepandensovirus |
| Penaeus monodon hepandensovirus 1 | NC_007218 | Parvoviridae; Densovirinae; Hepandensovirus |
