# Supplementary material for: An insight into the sialome, mialome and virome of the horn fly, Haematobia irritans
Source: BMC Genomics. 2019 Jul 29;20:616. doi: 10.1186/s12864-019-5984-7 (PMC6664567; doi:10.1186/s12864-019-5984-7)
Supplement: Supplementary file 3 — Link to supplemental spreadsheet. (DOCX 11 kb) [file 12864_2019_5984_MOESM3_ESM.docx]

Link to supplemental spreadsheet:

<https://s3.amazonaws.com/proj-bip-prod-publicread/transcriptome/Haematobia_irritans/2019/Hi-spreadsheet.zip>
